# Influence of Food Habits and Participation in a National Extracurricular Athletics Program on Body Weight within a Pair-Matched Sample of Polish Adolescents after One Year of Intervention—#goathletics Study

**DOI:** 10.3390/nu15245106

**Published:** 2023-12-14

**Authors:** Dominika Głąbska, Dominika Guzek, Dominika Skolmowska, Jakub Grzegorz Adamczyk, Hanna Nałęcz, Blanka Mellová, Katarzyna Żywczyk, Joanna Baj-Korpak, Krystyna Gutkowska

**Affiliations:** 1 Department of Dietetics, Institute of Human Nutrition Sciences, Warsaw University of Life Sciences (SGGW-WULS), 159C Nowoursynowska Street, 02-776 Warsaw, Poland; dominika_glabska@sggw.edu.pl (D.G.); dominika_skolmowska@sggw.edu.pl (D.S.); 2 Department of Food Market and Consumer Research, Institute of Human Nutrition Sciences, Warsaw University of Life Sciences (SGGW-WULS), 159C Nowoursynowska Street, 02-776 Warsaw, Poland; krystyna_gutkowska@sggw.edu.pl; 3 Department of Theory of Sport, Józef Piłsudski University of Physical Education, 34 Marymoncka Street, 00-968 Warsaw, Poland; jakub.adamczyk@awf.edu.pl; 4 Pedagogy and Psychology Department, Józef Piłsudski University of Physical Education, 34 Marymoncka Street, 00-968 Warsaw, Poland; hanna.nalecz@awf.edu.pl; 5 Nutrition, Health and Wellness Unit, Nestlé Polska S.A., 32 Domaniewska Street, 02-672 Warsaw, Poland; blanka.mellova@pl.nestle.com (B.M.); katarzyna.zywczyk@pl.nestle.com (K.Ż.); 6 Department of Physiotherapy, Faculty of Health Sciences, John Paul II University in Biala Podlaska, 95/97 Sidorska Street, 21-500 Biala Podlaska, Poland; j.baj-korpak@dyd.akademiabialska.pl

**Keywords:** body weight, body mass index, waist circumference, waist to height ratio, physical activity, food habits, adolescents

## Abstract

The COVID-19 pandemic reduced the physical activity level and commitment in adolescents, which has resulted in a body weight increase, and the World Health Organization (WHO) emphasizes that this negative trend must be now counteracted. The aim of this study was to assess the influence of food habits and participation in a national extracurricular athletics program (Athletics for All) on body weight within a pair-matched sample of Polish adolescents after one year of intervention. The #goathletics Study was conducted in a population of Polish adolescents within two pair-matched groups: 506 adolescents aged 10–14, including 281 female and 225 male adolescents, regularly participating in Athletics for All program for at least 9 months (one school year) (intervention group), and a pair-matched control group (matched taking into account city, gender, and age). The #goathletics Study included the assessment of the body weight, which was conducted based on the growth reference charts for Body Mass Index (BMI), and waist circumference to verify central fatness. Athletics for All participation and food habits were verified as determinants of body weight, while food habits were assessed using the validated Adolescents’ Food Habits Checklist (AFHC). While compared the intervention group and control group, statistically significant differences were observed for body weight, BMI, and waist circumference, both for crude and relative values, with adolescents participating in the Athletics for All program presenting a lower risk of excessive body weight and central fatness (*p* < 0.05), while, compared to sub-groups stratified by AFHC score, no statistically significant differences in general anthropometric characteristics were observed (*p* > 0.05). While the body weight centile, height centile, BMI centile and waist-to-height ratio (WHtR) were assessed as the resultant variables, it was revealed that participation in the Athletics for All program is the only influencing factor in multi-factor analysis of variance (ANOVA) for body weight centile (*F* = 21.44; *p* < 0.0001) and BMI centile (*F* = 47.98; *p* < 0.0001), but for height centile and WHtR, none of the assessed factors influenced these variables. It was concluded that regularly participating in the Athletics for All program for at least 9 months was the only determinant of a lower risk of excessive body weight in adolescents, with declared food habits and gender not being significant.

## 1. Introduction

The COVID-19 pandemic has triggered adverse effects in multiple areas of adolescents’ lives, including their physical health and fitness, mental health, relationships, and education, causing consequences for the so-called ‘COVID-19 generation’ that may be predicted for the following years [1]. National Polish studies of adolescents revealed that in the period of the COVID-19 pandemic, physical activity [2] and the self-regulation of eating behaviors were decreased [3], and as a result, worsened food habits were observed [4]. Similarly, for other countries, systematic reviews indicated a general decrease in the physical activity of adolescents during the pandemic, which was observed mainly during the first 1.5 years of the pandemic [5], and which was associated with decreased leisure time activity, decreased well-being, changed eating habits, and increased frequency of obesity, anxiety, and depression [6]. Such obvious negative consequences in terms of adolescents’ physical activity resulted from the implementation of various strategies in order to reduce virus transmission, including school closures, the close-down of leisure activities, the suspension of physical education classes at schools, and the general confinement of individuals [7].

Taking this into account, after the COVID-19 pandemic, it was necessary to change the behaviors of adolescents and introduce physical activity, in order to resume physical activity at least on the pre-pandemic level, if not even higher [8]. It may be especially beneficial for children with excessive body weight, while a recent systematic review by La Fauci et al. [9] indicated significant weight increases among children and adolescents during the pandemic, caused by higher consumption of hypercaloric junk food high in sugar, accompanied by reduced physical activity.

In order to promote physical activity in a population of children, there is a need to develop effective programs, which according to an umbrella review by Mannocci et al. [10], should be school-based and long-term, involving teachers and the support of families. According to a systematic review and meta-analysis by Jacob et al. [11], school-based interventions, including those focusing on physical activity and nutrition, have the public health potential to lower BMI in adolescents, while multi-component interventions involving teachers and parents are a promising strategy. Similarly, a recent systematic review by Soares et al. [12] indicated that physical activity may improve body weight and body composition in school-age children and adolescents with excessive body weight. Considering the serious problem observed after the COVID-19 pandemic, the World Health Organization (WHO) is currently developing practice- and science-informed, people-centered guidelines on the integrated management of excessive body weight in adolescents using a primary health care approach [13].

In recent times, various research initiatives have been undertaken to understand physical activity behaviors both on international and national levels [14]. Among such initiatives summarizing the current state of physical activity on a national level, there are Report Cards. The data from national Report Cards from different countries are then compiled to form the Global Matrix of Physical Activity Report Cards [14,15,16], in which 10 indicators of physical activity behaviors are assessed, such as overall physical activity, organized sport and physical activity, and active transportation or sedentary behaviors, while every indicator is graded from A+ (which refers to succeeding with between 94 and 100% of children) to F (succeeding with less than <20% of children). The results of Poland’s 2022 Report Card on the physical activity of children and adolescents, verified as a part of the Global Matrix 4.0 project, revealed that the situation in Poland is alarming, with only one indicator graded as B+ (school indicator), and all other graded as C+, C, C−, or D (moderate to weak scores) [14]. In spite of the fact that before COVID-19, some programs were conducted in Poland to promote physical activity and following a healthy diet, and a positive influence on body weight was observed [15], the WHO emphasizes that the COVID-19 pandemic reduced the physical activity level and commitment, and this negative trend must be now counteracted [16].

Given the described problem of reduced engagement in physical activity and physical activity levels among adolescents, as well as deterioration in dietary habits and increased body weight, the aim of this study was to assess the influence of food habits and participation in a national extracurricular athletics program (Athletics for All, being a voluntary and free-of-charge extracurricular physical activity program conducted in Poland since 2014 and organized by the Polish Athletic Association) on body weight within a pair-matched sample of Polish adolescents after one year of intervention.

## 2. Materials and Methods

### 2.1. Ethical Background

The #goathletics Study was conducted with the cooperation of (1) the Institute of Human Nutrition Sciences, Warsaw University of Life Sciences (SGGW-WULS) (responsible for the assessment of body weight and food habits), (2) Józef Piłsudski University of Physical Education (responsible for assessment of physical fitness), (3) the Polish Athletic Association (responsible for Athletics for All training groups), and (4) the Nutrition, Health and Wellness Unit, Nestlé Polska S.A. (responsible for Athletics for All program as one of the founders). Within the #goathletics Study, a Polish national extracurricular athletics program (Athletics for All) is assessed [17,18,19]. 

The Athletics for All program (in Polish, Lekkoatletyka Dla Każdego–LDK) [20] is a voluntary and free-of-charge extracurricular physical activity program conducted in Poland since 2014. It is organized by the Polish Athletic Association, with its trainers responsible for the training groups, supported by the Ministry of Sport and Tourism and by Nestlé Polska S.A. within their mission to promote a balanced diet. In 2023, over 600 training groups were organized in all the regions of Poland, independent of the size of the city. The main element of the Athletics for All program is providing regular athletics training (3 sessions per week of 90 min of moderate- to high-intensity physical activity), which is supported by nutritional education.

The #goathletics Study was approved by the Ethics Committee of the Warsaw University of Life Sciences (09/2023), and all the procedures were based on the Declaration of Helsinki. All participants, as well as their parents/legal guardians, provided written informed consent for participation. 

### 2.2. #goathletics Study Population

The #goathletics Study was conducted in a population of Polish adolescents within 2 pair-matched sub-groups:

(1) A total of 506 adolescents aged 10–14, including 281 female adolescents and 225 male adolescents, regularly participating in the Athletics for All program for at least 9 months (one school year) (intervention group);
(2) A total of 506 adolescents aged 10–14, including 281 female adolescents and 225 male adolescents, participating neither in the Athletics for All program nor in any other physical activity program for at least 9 months (one school year) (control group).

The intervention group was recruited from all the voivodeships in Poland (being the basic administrative unit in Poland), while for each voivodeship, a medium-sized city was chosen, and within this city, a typical training group within the Athletics for All program. Based on the data of the Central Statistical Office in Poland [21], the proportion of children aged 10–14 in each voivodeship was taken into account, and the sample size for each voivodeship was settled to keep the national proportions. 

The inclusion criteria for the intervention group were as follows:

(1) Caucasian individuals (major race in Poland);
(2) Adolescents from the chosen typical training group (one typical training group per voivodeship, located in a medium-sized city) with the Athletics for All program introduced;
(3) Adolescents aged 10–14,
(4) Adolescents regularly participating in Athletics for All program for at least 9 months (one school year—measurements conducted in June 2023, adolescents participating in a program since September 2022);
(5) Female and male adolescents in similar proportions;
(6) Provided written informed consent to participate in the #goathletics Study;
(7) Provided written informed consent of parents for participation in the #goathletics Study.

The exclusion criteria for the intervention group were as follows:

(1) Adolescents participating in any other additional physical activity program other than regular physical education classes (at school or after school classes);
(2) Any motor functions or cognitive disability diagnosed;
(3) Any missing data.

The control group was recruited from the same cities as the intervention group (one medium-sized city for each voivodeship), while for each city, a control group was recruited from one school, but with the Athletics for All program never introduced. 

The inclusion criteria for the control group were as follows:

(1) Caucasian individuals (major race in Poland);
(2) Adolescents from the chosen school (one school per voivodeship, located in a medium-sized city) with Athletics for All program not introduced;
(3) Adolescents aged 10–14,
(4) Adolescents participating neither in the Athletics for All program, nor in any other physical activity program for at least 9 months (one school year);
(5) Female and male adolescents in similar proportions;
(6) Adolescents pair-matched with those from the intervention group;
(7) Provided written informed consent to participate in the #goathletics Study;
(8) Provided written informed consent of parents for participation in the #goathletics Study.

The exclusion criteria for the control group were as follows:

(1) Any motor functions or cognitive disability diagnosed;
(2) Any missing data.

Pair-matching was applied, taking into account the following variables:

(1) City (paired individuals from the same city);
(2) Gender (paired individuals of the same gender);
(3) Age (paired individuals of the same age with ± 1 year of difference allowed if not possible to find an individual of identical age).

The other variables, associated with anthropometric measures, physical fitness, or food habits, were not taken into account for pair-matching, as they were intended to be compared between the intervention and control group. If there were 2 or more potentially eligible individuals to be pair-matched, the individual to be pair-matched was randomly selected. 

**Figure 1:**
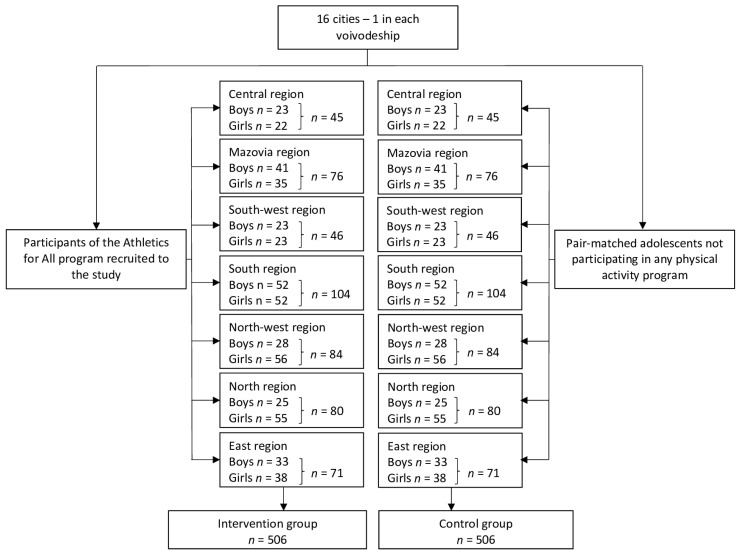
The distribution of the intervention group and control group within the geographical regions of Poland.

The distribution of the intervention group and control group within the geographical regions of Poland is presented in Figure 1. 

### 2.3. Assessment of Body Weight

The #goathletics Study included the assessment of body weight, which was conducted based on the Polish OLAF growth reference charts for Body Mass Index (BMI) [22].

The body weight and height of each adolescent were measured using a calibrated weighing scale (accuracy ± 0.1 kg) and a calibrated stadiometer (accuracy ± 0.5 cm), according to the commonly accepted and applied rules [23]. Based on the obtained values, BMI was calculated using the Quetelet equation [24] and assessed while being compared with OLAF growth reference charts, being specific for gender and age [25], using OLAF software 1.0 [22]. The obtained centile values were interpreted within the categories of underweight, normal weight, overweight, and obesity, based on the standard growth reference value cutoffs by the WHO, as follows: underweight for BMI < 5th percentile, normal weight for BMI of 5th–85th percentile, overweight for BMI of 85th–95th percentile, and obesity for BMI > 95th percentile [26].

Additionally, the waist circumference was assessed in order to define the central obesity tendency. The waist circumference was measured using a nonelastic flexible measuring tape (accuracy ± 0.5 cm) in the mid-abdominal area, in the horizontal plane midway between the lowest ribs and the iliac crest, according to the commonly accepted and applied rules [27]. Based on the obtained waist circumference and height of each adolescent, the waist-to-height ratio (WHtR) was calculated while dividing waist circumference by height [28] and interpreted within the categories of central fatness and no central fatness, based on the standard cutoffs, as follows: central fatness for WHtR > 0.500, and no central fatness for WHtR ≤ 0.500 [29].

### 2.4. Assessment of Food Habits

Food habits were assessed using the Adolescents’ Food Habits Checklist (AFHC) questionnaire developed for adolescents by Johnson et al. [30], being commonly applied in studies on adolescents in various countries [31,32], including Poland [33]. The previously developed Polish version of the AFHC was applied, after preparing it according to the recommendations of the WHO [34], including forward translation into Polish, backward translation into English, and final polishing by an expert panel. 

The AFHC was developed to assess healthy and unhealthy food habits, associated with purchasing (6 items), preparing (8 items), and consuming certain types of food (9 items). The highest possible score for the AFHC is 23 points, while the additional possibility of analysis is associated with dividing the studied group into sub-groups presenting healthy (fourth quartile of the score—the highest score), neutral (second and third quartile of the score), and unhealthy food habits (first quartile of the score—the lowest score), as recommended by Proserpio et al. [31].

### 2.5. Statistical Analysis

The assessed variables were body weight, BMI, and waist circumference, but body height was additionally analyzed in order to verify whether the compared samples may be considered representative and comparable. As there were no differences in body height, it was concluded that body weight, BMI, and waist circumference differences do not result from differences in bodily development processes but studied intervention or food habits.

The Shapiro–Wilk test was applied to verify the distribution of the data. An analysis of variance (ANOVA) was applied to compare groups with parametric distribution, and the Mann–Whitney U test and Kruskal–Wallis analysis of variance (ANOVA) were applied to compare groups with non-parametric distribution for all the conducted analysis. Additionally, the chi^2^ test was applied and a multi-factor ANOVA was applied to verify the most important determinants.

A level of significance of *p* ≤ 0.05 was interpreted as significant. The statistical analysis was conducted using Statistica 8.0 (Statsoft Inc., Tulsa, OK, USA) and Statgraphics Plus for Windows 4.0 (Statgraphics Technologies Inc., The Plains, VA, USA).

## 3. Results

The general anthropometric characteristics of the female and male adolescents within the #goathletics Study are presented in Table 1. Within the studied group, there are 83.5% normal-weight individuals, 4.2% underweight individuals, 8.4% overweight individuals, and 4.0% obese individuals. While comparing body weight, height, BMI, and waist circumference, gender-dependent differences were observed only for waist circumference, with higher circumference and WHtR for male compared to female participants (*p* < 0.05).

The general anthropometric characteristics of the intervention group and the control group of adolescents for female and male participants combined within the #goathletics Study are presented in Table 2. While comparing body weight, height, BMI, and waist circumference, the differences were observed for body weight, BMI, and waist circumference, both for crude and relative values. The higher values were observed for the control group of adolescents non-participating in the Athletics for All program than in those participating in the Athletics for All program (*p* < 0.05).

The general anthropometric characteristics of the female participants in the intervention group and control group of adolescents within the #goathletics Study are presented in Supplementary Table S1. While comparing body weight, height, BMI, and waist circumference, differences were observed for body weight, BMI, and waist circumference, both for crude and relative values. Higher values were observed in the control group of female adolescents not participating in the Athletics for All program than in those participating in the Athletics for All program (*p* < 0.05).

The general anthropometric characteristics of the male participants in the intervention group and control group of adolescents within the #goathletics Study are presented in Supplementary Table S2. While comparing body weight, height, BMI, and waist circumference, differences were observed for body weight, BMI, and waist circumference, both for crude and relative values. Higher values were observed in the control group of male adolescents not participating in the Athletics for All program than in those participating in the Athletics for All program (*p* < 0.05).

The general anthropometric characteristics of the intervention group and control group combined for subgroups within the #goathletics Study stratified by the AFHC score quartile are presented in Table 3. When comparing body weight, height, BMI, and waist circumference, no differences between the sub-groups of the food habit score were observed for any of them.

The general anthropometric characteristics of the intervention group for subgroups within the #goathletics Study stratified by the AFHC score quartile are presented in Supplementary Table S3. When comparing body weight, height, BMI, and waist circumference, no differences between the sub-groups of the food habit score were observed for any of them.

The general anthropometric characteristics of the control group for subgroups within the #goathletics Study stratified by the AFHC score quartile are presented in Supplementary Table S4. When comparing body weight, height, BMI, and waist circumference, no differences between the sub-groups of the food habit score were observed for any of them.

The interpretations of the general anthropometric characteristics of the female and male adolescents within the #goathletics Study are presented in Table 4. When comparing the central fatness risk, it was higher for male adolescents than for female ones (*p* = 0.0195).

The interpretations of the general anthropometric characteristics of the intervention group and control group of adolescents within the #goathletics Study are presented in Table 5. When comparing the excessive body weight and central fatness risk, they were higher in the control group of adolescents not participating in the Athletics for All program than in those participating in the Athletics for All program (*p* < 0.05).

The interpretations of the general anthropometric characteristics of adolescents for subgroups within the #goathletics Study stratified by the AFHC score quartile are presented in Table 6. When comparing the excessive body weight and central fatness risk, differences between the sub-groups of the food habit score were not observed.

Based on the one-factor analysis, it was revealed that the strongest predictor of the general anthropometric characteristics of adolescents seems to be participation in the Athletics for All program (in a comparison between the intervention group and control group). In order to confirm, it was verified within multi-factor ANOVA with gender (female vs. male participants), participation in the Athletics for All program (intervention group vs. control group), and Adolescents’ Food Habits Checklist (AFHC) score quartile (lowest score vs. average score vs. highest score). When the body weight centile, height centile, BMI centile, and WHtR were assessed as the resultant variables, it was revealed that participation in Athletics for All program is the only influencing factor within multi-factor ANOVA for body weight centile (*F* = 21.44; *p* < 0.0001) and BMI centile (*F* = 47.98; *p* < 0.0001), but for height centile and WHtR, none of the assessed factor influenced these variables.

## 4. Discussion

The conducted study confirmed the significant influence of physical activity program participation on body weight and excessive body weight risk. Interestingly, neither gender nor food habits were revealed to be as important determinants as physical activity program participation. 

In general, it is observed that in the majority of high- and upper-middle-income countries, the risk of obesity is higher in male than female adolescents aged 5–19 [35]. It is suggested to result from differences in obesogenic environment exposure and vulnerability, accompanied by differences in responses to conducted interventions [36]. It is stated that correct body weight perception is increasing in female adolescents but decreasing in male adolescents, while in female adolescents, there is a higher increase in the underestimation of weight status and a decrease in its overestimation compared to male individuals [37]. It corresponds with the higher waist circumference in male than female adolescents in the conducted study, resulting in a higher central fatness risk, but for BMI, statistically significant differences were not observed. Moreover, in the multi-factor analysis, gender was no longer an important factor influencing body weight in adolescents, with only physical activity program participation indicated as a significant determinant.

No statistically significant effect of eating habits (by AFHC score) on body weight in both observed groups of adolescents—participating and not participating in the extracurricular physical activity program—is the most important result of this study. Similar observations were formulated in the study by Taleb and Itani [38], as no association between the AFHC and overweight or obese BMI status was stated in a group of normal body weight and excessive body weight adolescents aged 14–19 years from Lebanon. The chosen tool is stated to be a valid and useful [30], while the results are associated with nutrition literacy [39], and scores for purchasing, preparing, and consuming within the AFHC are correlated [40]. 

In general, it is well known that food habits are associated with the risk of excessive body weight. The systematic review and dose–response meta-analysis of prospective studies by Schlesinger et al. [41] indicated that there are specific food product groups which are associated with the risk of excessive body weight, including inverse associations for whole grains, fruit, nuts, legumes, and fish, as well as positive associations for refined grains, red meat, and sugar-sweetened beverages. The systematic review and meta-analysis by Dakin et al. [42] indicated the influence of eating behavior traits which may predict energy intake and BMI, with susceptibility to hunger and binge eating as the strongest predictors of energy intake, as well as disinhibition as the strongest predictor of BMI. Taking this into account, it may be indicated that AFHC may not be a tool sensitive enough to reveal the associations with BMI, as AFHC focuses on specific choices available to adolescents [30], but their food habits result not only from their own food choices but also from the food choices of their parents and the other environmental determinants of their diet. Parents do influence the preferences and eating behaviors of their children, by influencing home food availability and acting as models of eating behaviors [43]. Additionally, the influence of peers is the other diet determinant, as adolescents commonly eat together, which means that not only individual food habits should be taken into account, but also food habits within a group, and a systematic review by Chung et al. [44] even indicated a need for group-based interventions to prevent excessive body weight in adolescents.

The other issue that may have interfered with the observed results is nutritional education, which was applied within the physical activity program; i.e., participants of the program, even if better food habits were not observed, have been educated, and adolescents not participating in the program, even if better food habits were observed, have not been educated. This means that the observed influence of the physical activity program in fact was an influence of the physical activity program combined with nutritional education. Such observation is supported by the results of the study comprising a systematic review and meta-analysis by Godoy-Cumillaf et al. [45], which studied the effect of physical activity and diet interventions on BMI in Latin American children and adolescents and revealed that if physical activity was combined with a diet, a statistically significant effect on BMI was observed. The presented observations are especially important if, in general, the educational interventions caused only a short-term influence on nutritional knowledge, while the long-term influence was low [46].

Last but not least, it must be emphasized that, in the present study, the physical activity program was the major determinant of body weight in adolescents. It is confirmed by the results of the systematic review by Lehmann et al. [47], indicating that regular physical activity can help prevent obesity. Similarly, the systematic review conducted by Ruotsalainen et al. [48] on interventions in excessive body weight revealed that the most valuable for body weight reduction are supervised interventions (as the one applied within the presented study). It is also confirmed by the results of the systematic review and meta-analysis by Moeini et al. [49], which specified the body weight reduction in case of adolescents as 1.02 kg (95% CI; reduction of 0.22–4.79 kg). Such results correspond with the observations from the present study—with a lower risk of excessive body weight and central fatness in the intervention group than in the control one. It indicates that the conducted physical activity program, combined with nutritional education, may be effective when conducted with adolescents. However, an especially important conclusion must be indicated from the COVID-19 perspective, as the possibility to counteract a negative trend of reduced physical activity level and commitment accompanied by increasing body weight [19] was proven.

In spite of the fact that the conducted study revealed important observations associated with the influence of the physical activity program on body weight and excessive body weight risk in a population of adolescents, some limitations of the study must be indicated. The most important limitation results from the applied method for measuring excessive body weight risk, namely BMI, while the assessment of body composition, including the share of the fat tissue, would allow us to obtain a deeper assessment. As the study was conducted on a national sample of adolescents from various cities and regions of Poland, such an assessment was straitened, especially in the period directly after the COVID-19 pandemic, while some restrictions were still in force in Poland, but the analyses of body composition conducted within the #goathletics Study in previous years correspond to the current observations [17].

## 5. Conclusions

It was concluded that regularly participating in the Athletics for All program for at least 9 months was the only determinant of lower risk of excessive body weight in adolescents, with declared food habits and gender not significant.

## Figures and Tables

**Table 1:** The general anthropometric characteristics of the female and male adolescents within the #goathletics Study.

		Total (*N* = 1012)		Female Participants (*N* = 562)		Male Participants (*N* = 450)		*p* **
		Mean (SD)	Median (Min–Max)	Mean (SD)	Median (Min–Max)	Mean (SD)	Median (Min–Max)	
Body weight	kg	45.08 (10.27)	44 * (23–92)	44.75 (9.81)	44 * (23–92)	45.5 (10.81)	44 * (23–88.8)	0.4811
	Centile	49.44 (27.6)	49 * (0–137)	49.48 (27.55)	49 * (0–137)	49.38 (27.71)	50 * (0–99)	0.9986
Height	cm	155.02 (9.4)	155 * (126.5–188.5)	154.6 (8.4)	155 (126.5–186)	155.53 (10.5)	155 * (126.5–188.5)	0.4772
	Centile	51.33 (29.46)	52 * (0–100)	50.69 (29.44)	51.5 * (0–100)	52.14 (29.49)	52 * (0–100)	0.4322
BMI	kg/m^2^	18.58 (2.94)	18.11 * (12.94–35)	18.56 (2.9)	18.11 * (12.94–35)	18.61 (2.99)	18.12 * (13.32–31.24)	0.8496
	Centile	48.64 (27.56)	48 * (0–100)	49 (27.15)	47.5 * (0–100)	48.19 (28.09)	48 * (0–99)	0.6401
Waist circumference	cm	66.28 (8.74)	65 * (29.67–100.17)	64.89 (8.45)	63.33 * (29.67–100.17)	68 (8.81)	66.75 * (51–96.33)	<0.0001
	WHtR	0.43 (0.14)	0.42 * (0.2–3.6)	0.43 (0.17)	0.41 * (0.2–3.6)	0.44 (0.08)	0.43 * (0.33–1.76)	<0.0001

* non-parametric distribution (*p* < 0.05; Shapiro–Wilk test); ** Mann–Whitney U test; BMI—Body Mass Index; WHtR—waist-to-height ratio.

**Table 2:** The general anthropometric characteristics of the intervention group and control group for female and male participants combined within the #goathletics Study.

		Intervention Group (*N* = 506, Including *N* = 281 Female and *N* = 225 Male)		Control Group (*N* = 506, Including *N* = 281 Female and *N* = 225 Male)		*p* **
		Mean (SD)	Median (Min–Max)	Mean (SD)	Median (Min–Max)	
Body weight	kg	43.6 (9.21)	43 * (24.6–88.8)	46.56 (11.04)	45 * (23–92)	<0.0001
	Centile	45.34 (25.47)	44.5 * (1–99)	53.53 (29.04)	55 * (0–137)	<0.0001
Height	cm	155.42 (9.63)	155 * (126.5–188.5)	154.62 (9.16)	154.5 * (130–186)	0.1710
	Centile	52.5 (28.86)	54 * (0–100)	50.16 (30.02)	51 * (0–100)	0.2037
BMI	kg/m^2^	17.88 (2.26)	17.65 * (12.94–28.34)	19.29 (3.34)	18.79 * (13–35)	<0.0001
	Centile	42.47 (24.94)	41 * (0–98)	54.81 (28.68)	58 * (1–100)	<0.0001
Waist circumference	cm	64.83 (7.74)	64 * (45–96.33)	67.73 (9.44)	66 * (29.67–100.17)	<0.0001
	WHtR	0.43 (0.17)	0.41 * (0.31–3.6)	0.44 (0.08)	0.43 * (0.2–1.76)	<0.0001

* non-parametric distribution (*p* < 0.05; Shapiro–Wilk test); ** Mann–Whitney U test; BMI—Body Mass Index; WHtR—waist-to-height ratio.

**Table 3:** The general anthropometric characteristics of the intervention group and control group combined for subgroups within the #goathletics Study stratified by the Adolescents’ Food Habits Checklist (AFHC) score quartile.

		Lowest Score for Food Habits (Q1 for AFHC) (*N* = 253, Including *N* = 123 Female and *N* = 130 Male)		Average Score for Food Habits (Q2 and Q3 for AFHC) (*N* = 506, Including *N* = 287 Female and *N* = 219 Male)		Highest Score for Food Habits (Q4 for AFHC) (*N* = 253 Including *N* = 152 Female and *N* = 101 Male)		*p* **
		Mean (SD)	Median (Min–Max)	Mean (SD)	Median (Min–Max)	Mean (SD)	**Median (Min–Max)**	
Body weight	[kg]	45.24 (10.77)	44 * (23–88.8)	45.18 (9.92)	44 * (25.4–88)	44.74 (10.47)	43.6 * (23–92)	0.7208
	Centile	48.83 (28.49)	50 * (0–99)	49.9 (27.5)	49 * (1–100)	49.11 (27.01)	48 * (0–137)	0.8296
Height	[cm]	155.44 (10.04)	155 (130–181)	154.79 (9.31)	154.5 * (127.5–188.5)	155.05 (8.93)	155 (126.5–180)	0.7414
	Centile	52.14 (29.73)	51 * (1–100)	50.44 (29.27)	51 * (0–100)	52.32 (29.61)	56 * (0–99)	0.5977
BMI	[kg/m^2^]	18.52 (2.97)	17.93 * (13.61–28.34)	18.71 (2.97)	18.31 * (13.32–35)	18.4 (2.83)	18.07 * (12.94–33.79)	0.3752
	Centile	47.01 (29.02)	45 * (1–98)	49.94 (27.56)	49 * (0–100)	47.67 (26)	46 * (0–100)	0.3199
Waist circumference	[cm]	66.42 (8.9)	64.83 * (52.33–96.33)	66.63 (8.68)	65 * (44.33–96)	65.42 (8.69)	64.33 * (29.67–100.17)	0.2256
	WHtR	0.44 (0.16)	0.42 * (0.33–2.51)	0.43 (0.05)	0.42 * (0.28–0.62)	0.43 (0.21)	0.42 * (0.2–3.6)	0.2299

* non-parametric distribution (*p* < 0.05; Shapiro–Wilk test); ** Kruskal–Wallis ANOVA; BMI—Body Mass Index; WHtR—waist-to-height ratio.

**Table 4:** The interpretations of the general anthropometric characteristics of the female and male adolescents within the #goathletics Study.

		Total (*N* = 1012)	Female Participants (*N* = 562)	Male Participants (*N* = 450)	*p* *
BMI	Underweight	42 (4.2%)	24 (4.3%)	18 (4.0%)	0.8941
	Normal weight	845 (83.5%)	471 (83.8%)	374 (83.1%)	
	Overweight	85 (8.4%)	44 (7.8%)	41 (9.1%)	
	Obesity	40 (4.0%)	23 (4.1%)	17 (3.8%)	
WHtR	No central fatness	915 (90.4%)	519 (92.3%)	396 (88.0%)	0.0195
	Central fatness	97 (9.6%)	43 (7.7%)	54 (12.0%)	

* chi^2^ test; BMI—Body Mass Index; WHtR—waist-to-height ratio.

**Table 5:** The interpretations of the general anthropometric characteristics of the intervention group and control group of adolescents within the #goathletics Study.

		Intervention Group (*N* = 506, Including *N* = 282 Female and *N* = 225 Male)	Control Group (*N* = 506, Including *N* = 282 Female and *N* = 225 Male)	*p* *
BMI	Underweight	23 (4.5%)	19 (3.8%)	<0.0001
	Normal weight	453 (89.5%)	392 (77.5%)	
	Overweight	25 (4.9%)	60 (11.9%)	
	Obesity	5 (1.0%)	35 (6.9%)	
WHtR	No central fatness	480 (94.9%)	435 (86%)	<0.0001
	Central fatness	26 (5.1%)	71 (14%)	

* chi^2^ test; BMI—Body Mass Index; WHtR—waist-to-height ratio.

**Table 6:** The interpretations of the general anthropometric characteristics of adolescents for subgroups within the #goathletics Study stratified by the Adolescents’ Food Habits Checklist (AFHC) score quartile.

		Lowest Score for Food Habits (Q1 for AFHC) (*N* = 253, Including *N* = 123 Female and *N* = 130 Male)	Average Score for Food Habits (Q2 and Q3 for AFHC) (*N* = 506, Including *N* = 287 Female and *N* = 219 Male)	Highest Score for Food Habits (Q4 for AFHC) (*N* = 253, Including *N* = 152 Female and *N* = 101 Male)	*p* *
BMI	Underweight	17 (6.7%)	16 (3.2%)	9 (3.5%)	0.2573
	Normal weight	206 (81.1%)	421 (83.5%)	218 (85.8%)	
	Overweight	19 (7.5%)	48 (9.5%)	18 (7.1%)	
	Obesity	12 (4.7%)	19 (3.8%)	9 (3.5%)	
WHtR	No central fatness	236 (92.9%)	455 (90.3%)	236 (92.9%)	0.3192
	Central fatness	18 (7.1%)	49 (9.7%)	18 (7.1%)	

* chi^2^ test; BMI—Body Mass Index; WHtR—waist-to-height ratio.

## Data Availability

The data presented in this study are available on request from the corresponding author. The data are not publicly available due to ethical restrictions.

## Supplementary Materials

The following supporting information can be downloaded at: https://www.mdpi.com/article/10.3390/nu15245106/s1, Table S1. The general anthropometric characteristics of the female participants in the intervention group and control group within the #goathletics Study. Table S2. The general anthropometric characteristics of the male participants in the intervention group and control group within the #goathletics Study. Table S3. The general anthropometric characteristics of the intervention group for subgroups within the #goathletics Study stratified by the Ad-olescents’ Food Habits Checklist (AFHC) score quartile. Table S4. The general anthropometric characteristics of the control group for subgroups within the #goathletics Study stratified by the Adolescents’ Food Habits Checklist (AFHC) score quartile.
