# Supplementary material for: Influence of Food Habits and Participation in a National Extracurricular Athletics Program on Body Weight within a Pair-Matched Sample of Polish Adolescents after One Year of Intervention—#goathletics Study
Source: Nutrients. 2023 Dec 14;15(24):5106. doi: 10.3390/nu15245106 (PMC10745295; doi:10.3390/nu15245106)
Supplement: Supplementary file 1 [file nutrients-15-05106-s001.zip › nutrients-2713554-supplementary.pdf]

nutrients

MDPI

Supplementary Material

Influence of Food Habits and Participation in a National Extracurricular Athletics Program on Body Weight within a Pair-Matched Sample of Polish Adolescents after One Year of Intervention—#goathletics Study

Dominika Głąbska [1], Dominika Guzek [2,*], Dominika Skolmowska [1], Jakub Grzegorz Adamczyk [3], Hanna Nałęcz [4], Blanka Mellová [5], Katarzyna Żywczyk [5], Joanna Baj-Korpak [6] and Krystyna Gutkowska [2]

[1] Department of Dietetics, Institute of Human Nutrition Sciences, Warsaw University of Life Sciences (SGGW-WULS), 159C Nowoursynowska Street, 02-776 Warsaw, Poland; dominika_glabska@sggw.edu.pl (D.G.); dominika_skolmowska@sggw.edu.pl (D.S.)
[2] Department of Food Market and Consumer Research, Institute of Human Nutrition Sciences, Warsaw University of Life Sciences (SGGW-WULS), 159C Nowoursynowska Street, 02-776 Warsaw, Poland; krystyna_gutkowska@sggw.edu.pl
[3] Department of Theory of Sport, Józef Piłsudski University of Physical Education, 34 Marymoncka Street, 00-968 Warsaw, Poland; jakub.adamczyk@awf.edu.pl
[4] Pedagogy and Psychology Department, Józef Piłsudski University of Physical Education, 34 Marymoncka Street, 00-968 Warsaw, Poland; hanna.nalecz@awf.edu.pl
[5] Nutrition, Health and Wellness Unit, Nestlé Polska S.A., 32 Domaniewska Street, 02-672 Warsaw, Poland; blanka.mellova@pl.nestle.com (B.M.); katarzyna.zywczyk@pl.nestle.com (K.Ż.)
[6] Department of Physiotherapy, Faculty of Health Sciences, John Paul II University in Biala Podlaska, 95/97 Sidorska Street, 21-500 Biala Podlaska, Poland; j.baj-korpak@dyd.akademiabialska.pl
* Correspondence: dominika_guzek@sggw.pl; Tel.: +48-22-593-71-34

Supplementary Table S1. The general anthropometric characteristics of the female participants in the intervention group and control group within the #goathletics Study.

		**Female participants in intervention group (*N* = 281)**		**Female participants in control group (*N* = 281)**		***p*********
		Mean (SD)	Median (min-max)	Mean (SD)	Median (min-max)	
Body weight	kg	43.35 (8.36)	43.8* (25.4-79)	46.15 (10.9)	44.2* (23-92)	0.0114
	Centile	45.72 (25.32)	46* (1-99)	53.23 (29.17)	55* (0-137)	0.0018
Height	cm	155 (8.44)	155* (126.5-174.5)	154.2 (8.36)	154 (132-186)	0.1274
	Centile	52.33 (29.12)	55* (0-100)	49.04 (29.72)	50* (1-100)	0.1881
BMI	kg/m^2	17.9 (2.19)	17.75* (12.94-27.34)	19.23 (3.34)	18.74* (13-35)	<0.0001
	Centile	43.22 (24.56)	42* (0-98)	54.78 (28.4)	59* (1-100)	<0.0001
Waist circumference	cm	63.67 (7.3)	63* (45-86.67)	66.11 (9.32)	64.33* (29.67-100.17)	0.0035
	WHtR	0.43 (0.23)	0.41* (0.31-3.6)	0.43 (0.06)	0.42* (0.2-0.62)	0.0007

* non-parametric distribution ($p < 0.05$; Shapiro-Wilk test); ** Mann-Whitney U test; BMI – Body Mass Index; WHtR – waist-to-height ratio.

Supplementary Table S2. The general anthropometric characteristics of the male participants in the intervention group and control group within the #goathletics Study.

		**Male participants in intervention group (*N* = 225)**		**Male participants in control group (*N* = 225)**		***p*********
		Mean (SD)	Median (min-max)	Mean (SD)	Median (min-max)	
Body weight	kg	43.92 (10.18)	42.6* (24.6-88.8)	47.08 (11.21)	45.2* (23-79.6)	0.0013
	Centile	44.86 (25.7)	43* (1-98)	53.91 (28.94)	56* (0-99)	0.0005
Height	cm	155.93 (10.94)	155* (126.5-188.5)	155.13 (10.06)	155 (130-184)	0.6327
	Centile	52.72 (28.6)	52* (1-100)	51.56 (30.4)	52* (0-100)	0.6866
BMI	kg/m^2	17.85 (2.35)	17.53* (13.32-28.34)	19.37 (3.35)	18.85* (13.61-31.24)	<0.0001
	Centile	41.54 (25.42)	39* (0-97)	54.84 (29.09)	56* (1-99)	<0.0001
Waist circumference	cm	66.27 (8.03)	64.67* (51-96.33)	69.74 (9.21)	69* (51.67-96.33)	<0.0001
	WHtR	0.43 (0.04)	0.42* (0.33-0.56)	0.46 (0.1)	0.44 (0.35-1.76)	<0.0001

* non-parametric distribution ($p < 0.05$; Shapiro-Wilk test); ** Mann-Whitney U test; BMI – Body Mass Index; WHtR – waist-to-height ratio.

Supplementary Table S3. The general anthropometric characteristics of the intervention group for subgroups within the #goathletics Study stratified by the Adolescents' Food Habits Checklist (AFHC) score quartile.

		Lowest score for food habits (Q1 for AFHC) (*N* = 114, including *N* = 64 female and *N* = 50 male)		Average score for food habits (Q2 and Q3 for AFHC) (*N* = 242, including *N* = 134 female and *N* = 108 male)		Highest score for food habits (Q4 for AFHC) (*N* = 150, including *N* = 83 female and *N* = 67 male)		*p***
		Mean (SD)	Median (min-max)	Mean (SD)	Median (min-max)	Mean (SD)	Median (min-max)	
Body weight	[kg]	44.51 (10.48)	42.6* (29-88.8)	43.57 (9.06)	43.8* (25.4-73.2)	42.76 (8.08)	43* (24.6-69)	0.7572
	Centile	45.87 (26.19)	47* (2-99)	44.75 (25.84)	43* (1-98)	45.98 (24.11)	47* (3-93)	0.8531
Height	[cm]	156.49 (10.36)	155.5 (132-181)	155.27 (9.63)	155 (127.5-188.5)	154.64 (8.82)	155 (126.5-180)	0.1796
	Centile	53.63 (29.35)	55* (1-100)	51.23 (28.64)	52* (0-100)	53.89 (28.92)	56* (0-99)	0.6219
BMI	[kg/m2]	17.98 (2.55)	17.59* (13.97-28.34)	17.9 (2.27)	17.69* (12.94-25.63)	17.73 (1.89)	17.75* (13.21-22.31)	0.9534
	Centile	41.54 (26.14)	41* (1-98)	42.7 (25.47)	41* (0-96)	42.94 (22.71)	42 (1-90)	0.7885
Waist circumference	[cm]	64.84 (8.2)	63* (52.33-96.33)	64.84 (7.9)	63.2* (45-86.67)	64.77 (6.96)	64.67* (48.33-83)	0.8788
	WHtR	0.43 (0.19)	0.41* (0.33-2.51)	0.42 (0.04)	0.41* (0.31-0.56)	0.44 (0.28)	0.42* (0.32-3.6)	0.3796

* non-parametric distribution ($p < 0.05$; Shapiro-Wilk test); ** analysis of variance (ANOVA)/ Kruskal–Wallis ANOVA; BMI – Body Mass Index; WHtR – waist-to-height ratio.

Supplementary Table S4. The general anthropometric characteristics of the control group for subgroups within the #goathletics Study stratified by the Adolescents' Food Habits Checklist (AFHC) score quartile.

		**Lowest score for food habits (Q1 for AFHC) (*N* = 139, including *N* = 59 female and *N* = 80 male)**		**Average score for food habits (Q2 and Q3 for AFHC) (*N* = 264, including *N* = 153 female and *N* = 111 male)**		**Highest score for food habits (Q4 for AFHC) (*N* = 103, including *N* = 69 female and *N* = 34 male)**		*p***
		Mean (SD)	Median (min-max)	Mean (SD)	Median (min-max)	Mean (SD)	Median (min-max)	
Body weight	[kg]	47.45 (11.9)	45* (23-92)	46.87 (10.63)	45* (26.4-88)	45.06 (10.89)	44* (23-74)	0.2477
	Centile	54.39 (29.42)	55* (0-137)	55.23 (27.89)	59* (1-100)	49.31 (30.66)	51* (0-98)	0.1809
Height	[cm]	155.43 (8.71)	155 (133-176)	154.56 (9.06)	154 (132-186)	153.91 (9.77)	154 (130-180)	0.6616
	Centile	49.98 (30.75)	45* (1-100)	50.81 (29.42)	52* (0-100)	49.06 (30.66)	46* (1-100)	0.8816
BMI	[kg/m2]	19.42 (3.48)	18.9* (13-33.79)	19.47 (3.3)	18.81* (13.78-35)	18.82 (3.25)	18.47* (13.61-27.99)	0.1103
	Centile	55.24 (28)	59* (1-100)	56.95 (27.76)	61.5* (2-100)	50.12 (30.77)	53* (1-98)	0.1263
Waist circumference	[cm]	66.83 (9.84)	65* (29.67-100.17)	68.17 (9.24)	66.67* (44.33-96)	67.74 (9.42)	66* (52.33-96.33)	0.2951
	WHtR	0.43 (0.06)	0.42* (0.2-0.61)	0.44 (0.06)	0.43* (0.28-0.62)	0.45 (0.13)	0.43* (0.35-1.76)	0.1512

* non-parametric distribution ($p < 0.05$; Shapiro-Wilk test); ** analysis of variance (ANOVA)/ Kruskal–Wallis ANOVA; BMI – Body Mass Index; WHtR – waist-to-height ratio
